# Association between Cardiopulmonary Capacity and Body Mass Composition in Children and Adolescents with High Body Weight: A Cross-Sectional Study

**DOI:** 10.3390/children9050647

**Published:** 2022-04-30

**Authors:** Agata Dobrowolska, Małgorzata Domagalska-Szopa, Andrzej Siwiec, Andrzej Szopa

**Affiliations:** 1 Department of Developmental Age Physiotherapy, Medical University of Silesia in Katowice, 40-751 Katowice, Poland; adobrowolska@sum.edu.pl (A.D.); mdomagalska@sum.edu.pl (M.D.-S.); 2 John Paul II Pediatric Center in Sosnowiec, 41-218 Sosnowiec, Poland; asivietz@wp.pl; 3 Department of Physiotherapy, Medical University of Silesia in Katowice, 40-751 Katowice, Poland

**Keywords:** cardiopulmonary exercise test (CPET), body mass composition, children, adolescents, high BMI

## Abstract

(1) Background: Excessive body weight is a global problem in the 21st century. Children and adolescents, in particular, are at risk. Recently, there has been an increasing interest in the relationship between aerobic capacity and body composition. Therefore, this study aimed to determine the association between the individual parameters of cardiopulmonary capacity obtained in cardiopulmonary exercise testing (CPET) and selected parameters of body mass composition in high-BMI children and children over the 85th percentile according to the WHO growth reference. (2) Materials and Method: The research included 100 children of school-age (7–15 years) with an excessive BMI, i.e., over the 85th percentile as per the WHO Growth Reference (BMI percentile 95.21 ± 4.65; Z-score BMI: 2.07 ± 0.94). The study consisted of three parts: anthropometric measurements, measurement of body mass composition using a body composition analyzer (TANITA MC-780 S MA) using the bioimpedance method, and a cardiopulmonary exercise test on a pediatric cycle ergometer (Corival Pediatric, Lode BV) using the Godfrey protocol; (3) Results: The correlation between BMI and fat mass (FM) was very high (rho = 0.83; *p* = 0.00) with moderate body fat percentage (BF%) (rho = 0.48; *p* = 0.00). There was a relevant correlation between the amount of fat-free mass in total body mass and cardiopulmonary capacity expressed as the absolute aerobic capacity (VO_2peak_) (rho = 0.55; *p* = 0.00). (4) Conclusions: In the case of children and youth with higher BMI, there was a correlation between the amount of fat-free mass in total body mass and cardiopulmonary capacity in terms of absolute aerobic capacity.

## 1. Introduction

Over the last 40 years, the number of overweight people has risen threefold [1]. A high body mass index (BMI) also concerns youth and children. Overweight body mass may be associated with disturbances in the development of youth cardiorespiratory fitness [2,3,4,5]. Being overweight during childhood increases obesity risk in adults and is responsible for metabolic diseases, hypertension, and degenerative joint disease [1,6,7]. 

For many years, there has been great interest in the relationship between physical efficiency and body mass in children and adolescents. Previous studies on age-related growth changes in the body mass parameters and their association with cardiorespiratory fitness (CRF) have given conflicting reports from strong correlation to no correlation between BMI and peak oxygen uptake (VO_2peak_). Several studies using the traditional interpretation of VO_2peak_ in ratio with body mass showed an inverse relationship between the ratio scaling VO_2peak_ with BMI in the pediatric population [2,3,4,5,8]. At the same time, other pediatric exercise studies based on multilevel allometric modeling of longitudinal data sets to elucidate the sex-specific development of VO_2peak_ have shown that ratio scaling of VO_2peak_ ignoring concurrent sex-specific changes with age and maturity status does not control for total BMI and may misinterpret the development of youth CRF [9,10,11,12]. 

Most studies focused on the correlation of physical efficiency (i.e., VO_2max_ or VO_2peak_) with anthropometric measurements such as age, height, weight, and above all, body mass index (BMI) [13,14,15,16,17,18,19]. Although BMI monitors changes in body mass, it has many inaccuracies [6,20,21]. First, BMI does not define particular body mass composition parameters (e.g., it does not diversify fat and lean mass). Second, it does not provide information on changes in the composition of a young individual during phases of intensive growth, where the increase in BMI is primarily a result of an increase in lean mass, i.e., fat-free mass (FFM). Third, it does not account for aberrant fat mass distribution limited to specific body locations [6,20].

Although a few studies have analyzed the relationship between direct cardiopulmonary capacity and body mass composition in the pediatric population, they usually have been conducted in an entire school children population [22,23], and/or used an estimated VO_2_ (max) from tests (e.g., Rockport Walk Fitness Test, Harvard Step Test, Cooper 12-min run test and 20 m shuttle run test (20m SRT) as a replacement for direct youth peak ˙VO assessment [2,5,11,24] or anthropometric measures (e.g., height, weight, BMI, waist circumference, hip circumference, and skinfold thickness) were used as a substitute of body mass composition [9,17,24].

Based on our previous study, where recognizing differences in cardiopulmonary capacity between overweight and obese children and adolescents in comparison to normal weight peers, we identified the relationship between VO_2peak_ and body weight in high BMI children we have decided to refine these relationships [25].

Therefore, this study aimed to determine the association between the individual parameters of cardiopulmonary capacity obtained in cardiopulmonary exercise testing (CPET) and selected parameters of body mass composition in high-BMI children and children over the 85th percentile according to the WHO growth reference [26]. We hypothesized that: (1) the best variable to describe the body size in relation to the cardiorespiratory fitness in overweight and obese children is fat-free mass, and (2) the lower the proportion of fat-free mass in the body, the lower VO_2peak_ in the population of children and adolescents with high BMI. 

## 2. Materials and Methods

The study design, protocol, and consent forms were designed in accordance with the Code of Ethics of the World Medical Association (Declaration of Helsinki). This study was approved by the Ethical Committee of the Medical University of Silesia (KNW/0022/KB1/38/18). Before the examination, parents or caregivers were informed about the course and risks of this study. Additionally, a written informed consent form was signed by the parents/caregivers. Adolescents from 12 to 15 years of age gave consent independently, as minors, in addition to their parents/legal representatives or guardians.

An a priori sample size analysis was performed to detect a large effect size with α as 0.05 and the power of the study (90–95% participants). 

The study comprised 100 school-aged children (7–15 years old) with a BMI above the 85th percentile (WHO Growth Reference) [26], 52 of them were overweight (BMI percentile 91.33 ± 3.17), and 48 were obese (BMI centile 99.35 ± 0.66). The participants were recruited from the Program for the Treatment of Overweight and Obese Children organized by a local Silesian pediatric rehabilitation center. The research inclusion criteria were as follows: (1) BMI over 85th percentile, for age and sex, according to the WHO growth reference; (2) Confirmed clinically healthy; (3) School-age 7–15 years; (4) Ability to understand and follow instructions; (5) Informed consent of a parent/caregiver.

The subjects were excluded from the study when: (1) children were diagnosed with metabolic syndrome, severe, chronic, cardiac, or neurological disease; or (2) occurrence of significant coordination and balance disorders that lead to falls (based on a detailed medical history). The characteristics of the participants are shown in Table 1.

### 2.1. Methods

The study consisted of three parts: (1) Anthropometric measurements; (2) Measure body mass composition; (3) Cardiopulmonary Exercise Test (CPET). The examination was performed by the same experienced examiner (physiotherapist) in a single experimental room between 8.00 and 11.00 h.

#### 2.1.1. Anthropometric Measurements

Bodyweight and height were among the anthropometric measurements performed. Tanita MC-780 S MA (Tanita Corporation, Tokyo, Japan) body composition analyzer was used to measure the body weight (kg), and the results were recorded with an accuracy of 100 g. All measurements were performed in the morning after a light breakfast. During the test, the children wore light clothing. Meanwhile, height was measured as the maximal distance from the highest point of the head to the floor using the Tanita HR-001 (Tanita Corporation, Tokyo, Japan) mobile height meter. During this measurement, the child’s feet were brought together; the arms were hanging freely along the body; and the upper back, buttocks, and heels were in contact with the meter. Both measurements were performed in the morning after a light breakfast. Based on the aforementioned measurements, the following indices were calculated:

(1) BMI: Calculated as weight divided by height squared (kg/m^2^)
(2) BMI z-score and BMI for age and sex percentile were calculated using the WHO AnthroPlus software, which provides the WHO 2007 references for school-age children and adolescents.

#### 2.1.2. Measure Body Mass Composition

A multi-frequency segmented body mass composition analyzer (TANITA MC-780 S MA) employing bioimpedance analysis (BIA) was used to examine body mass composition in children. The analysis of all body mass composition components took approximately 20 s. Age, sex, and height without a decimal number were entered into the analyzer. The standard was selected for all subjects. The analysis was performed in a barefoot position, with each child barefoot and dressed in underwear. For the purposes of this study, this segmental body mass composition analyzer measured body mass index, basal metabolic rate, body fat percentage, fat mass, fat-free mass, total body water, predicted muscle mass, impedance, bone mass, and phase angle (Table 2) [14,27,28,29,30,31,32,33,34,35,36].

#### 2.1.3. Cardiopulmonary Exercise Test (CPET)

MetaLyzer^®^ 3B with Breath-by-Breath technology (Cortex Biophysik GmbH, Leipzig, Germany) was used in the study in addition to the cycle ergometer (Corival Pediatric, Lode BV, Groningen, Netherlands). Every participant underwent the maximum progressive test CPET until the exhaustion point. CPET was performed as described and used in our previous using the Godfrey cycle ergometer protocol [37]. CPET started with 2 min rest, then 2 min warmup with 0 W workload, and then with a constantly increasing workload of 10 W (for patients with height under 120 cm), 15 W (for patients with a height from 120 to 150 cm), and 20 W (for patients with a height over 150 cm) every minute until planned exhaustion. The equipment was calibrated before every test, as per the manufacturer’s instructions. The seat’s height and the child’s position were individually selected for considering leg length and ride comfort. Each participant was informed of the requirement of a constant speed level (approximately 60 rpm). The acceptable revolutions per minute ranged from 55–65.

The following parameters of cardiopulmonary capacity (CPET) were recorded: heart rate (HR), breathing frequency (BF), peak oxygen consumption/maximal oxygen uptake (VO_2peak_), peak oxygen per body mass (VO_2peak/kg_), power output (W), minute ventilation volume (VE), ventilation equivalent for oxygen (VE/VO_2_), ventilation equivalent for carbon dioxide (VE/VCO_2_); and RER, defined as the ratio between carbon dioxide production. The CPET was considered finished when despite increasing the intensity of the exerciser, there was a VO_2max_ plateau.; RER was higher or equal to 1.1 and heart rate was higher than 85% of the maximum heart rate calculated for age [38]. The test was stopped if the child showed subjective symptoms of exhaustion or, despite verbal encouragement, could not maintain the required circulation frequency. The result was registered when an oxygen uptake plateau was reached, in the case of no rate stabilization of maximal VO_2_. was registered. When the VO_2max_ plateau was not maintained, VO_2peak_ was recorded.

#### 2.1.4. Statistical Analyses

Statistica 13.3 (by Tibco) software was used to perform the statistical analyses [39]. The Shapiro-Wilk test was performed to assess the normality of the distribution of variables. Descriptive statistics were calculated for the clinical characteristics of each group and presented in terms of mean, median, standard deviation, quartiles, minimum, and maximum values.

Spearman’s correlation was used to assess the correlation between BMI and selected body composition parameters. Additionally, the Spearman correlation was used to assess the correlation between body mass composition and parameters of cardiopulmonary capacity. Only the correlation rates with *p* < 0.05 were considered statistically significant. Correlation rates were calculated according to Altman recommendations: Rs < 0.2, poor; 0.21–0.4, low; 0.41–0.6, moderate; 0.61–0.8, high and 0.81–1, very high. Simple linear regression was used to describe BMI as a predictor of each cardiovascular capacity parameter.

## 3. Results

**Figure 1:**
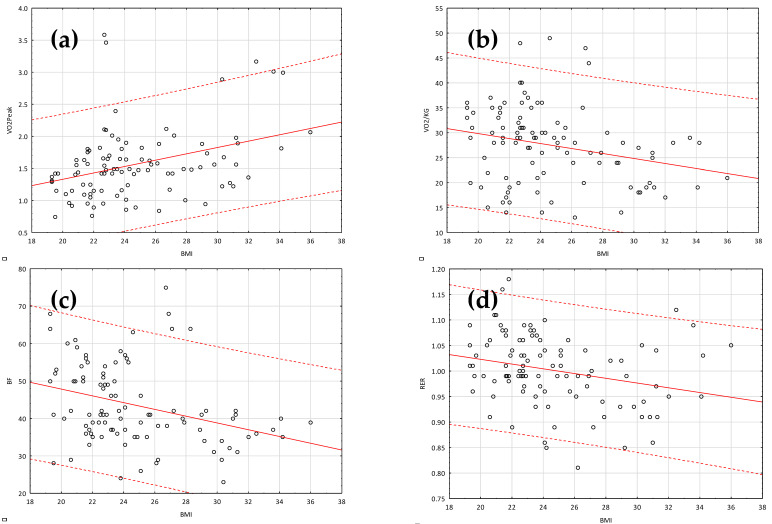
BMI as a predictor of VO_2peak_ (**a**) and VO_2peak/kg_ (**b**), BF (**c**), RER (**d**).

Table 3 summarizes body mass composition data (Tanita test). Data on cardiopulmonary capacity (CPET) in children with high BMI are presented in Table 4. In the case of BMI, the correlation with FM was very high, and BF% was moderate (Table 5). These results confirm an obvious relationship, which states that the higher the BMI, the higher the fat mass (expressed as the BF% rate and fat mass), and the higher the BMI, the higher the fat-free mass. Because basic parameters assessing cardiopulmonary capacity level are absolute aerobic capacity and relative aerobic capacity, the correlation between body composition parameters was carefully analyzed. An analysis of the results allowed us to observe the relevant correlations between the capacity parameters and BMI. A simple linear regression was calculated to predict BF, VO_2peak_, VO_2peak/kg_, and RER based on BMI (Table 6; Figure 1).

A significant correlation was observed between fat-free mass (FFM) and the level of cardiopulmonary capacity in terms of peak power output (W), minute ventilation volume (WE), and breathing frequency (BF) (Table 7). The higher all capacity parameters, including cardiopulmonary capacity, the higher the FFM. There was a relevant correlation between absolute aerobic capacity and relative aerobic capacity and fat-free mass. These inverse correlations showed that the higher the fat mass, the lower the oxygen capacity per 1 kg.

## 4. Discussion

This study is part of a broader research project that involves a mutual correlation between capacity assessment and body composition of children and youth with high BMI. The first scientific reports related to this research project involved assessing high BMI children’s oxygen uptake and were presented in our previous article “Cardiopulmonary Capacity in Overweight and Obese Children and Adolescents: A Cross-Sectional Study.” A total of 150 children participated in this research (partly participants of the current study) in the Program for the Treatment of Overweight and Obese Children organized by a local Silesian pediatric rehabilitation center. Studies have found that obese children and adolescents present lower relative aerobic capacity in relation to body weight compared to their peers with normal body weight and overweight children. The obtained results agreed with those of other authors who reported that children with high BMI (over the 85th percentile according to the WHO Growth Reference) had reduced VO_2peak_.

It is common knowledge that overweight and obesity are pathological states of adipose tissue accumulation that affect the cardiometabolic state, even among the youngest children. As already indicated above, although BMI is considered the standard monitoring tool for overweight and obese children, it does not distinguish fat mass from fat-free mass in body composition, and so it does not show reliable results [40,41]. Hence other rates and means are needed to assess children and youth nutrition states, especially those who are overweight and obese [42].

Therefore, body composition is becoming more important in diagnosing children’s diseases associated with high BMI in overweight and obese children. Recently, bioelectric impedance analysis (BIA) has become the most popular body composition assessment method. It is a simple, non-invasive tool for measuring and monitoring individual changes in body fat, lean mass, and hydration status. Because of the use of BIA in the above research, it was possible not only to calculate BMI but also to determine the content of body mass components, such as basal metabolic rate, predicted muscle mass, bone mass, and phase angle.

Fat mass in body mass per kg and percentage were accordingly 19.72 ± 7.63 kg and 31.89 ± 5.65%, and also fat-free mass (approximately 39.90 ± 10.74 kg) and average BMR was (6388.63 ± 1260 kJ) were similar to those presented by others [1,7,13,27,30,43,44,45,46,47,48,49]. The body composition results obtained in our study, were not different from other research results, showing that the examined population with a high BMI is typical, which substantiates the correlation between body mass components and cardiopulmonary capacity rates.

The main discovery of this study was the recognition of the correlation between the level of cardiopulmonary capacity and body mass composition in children with a high BMI. To the best of our knowledge, there are few studies on the correlation between cardiopulmonary capacity rates measured by CPET and particular body composition parameters specified by BIA in children with a high BMI. The analysis of these correlations proved that the lower the cardiopulmonary capacity of the subjects, expressed in absolute aerobic capacity and relative aerobic capacity, the higher their BMI. On the other hand, the analysis considering body mass components showed that the strongest correlation was between fat-free mass (FFM) and cardiopulmonary capacity level expressed in maximum oxygen uptake (VO_2peak_), peak power output (W), and minute ventilation volume (VE) and breathing frequency (BF). These results are compatible with previous research stating that fat-free mass level is the main factor affecting VO_2peak_ [17,23]. Quoted research were based on other methods of measuring body composition; anthropometric measurements used were: skinfold thickness, waist circumference, and hip circumference; VO_2max_ and cardiorespiratory efficiency (CRF) were determined on the basis of the Rockport Walk Fitness. On the other hand, in the second study, the measurement of mass body composition was measured by the dual-energy X-ray absorptiometry method and bioimpedance analysis and an exercise test on a bicycle ergometer with the analysis of respiratory gases were used [17,23].

In addition, research results related to healthy populations and students (aged 7–17 years) revealed that it was associated with FFM [15,50]. Moreover, previous studies [46,48,49] have confirmed that our results were related to inversely proportional correlations between VO_2peak/kg_, BMI, and BF%. Previous and presented research indicates that another factor responsible for a lower cardiopulmonary capacity of overweight children and youth is the fat mass (FM) and BF%. The percentage of body fat (% BF) is key to preventing overweight and obesity, and the VO_2peak_ result contributes to this. Individuals with excess body weight have reduced VO_2peak_ results, which clearly shows that the percentage of adipose tissue significantly impacts aerobic capacity. It should be noted that children and adolescents with excess BF are 2.2 times more likely to be at risk of cardiovascular disease and that adipose tissue itself is a modifiable and variable risk factor. It has also been reported that the incidence of high% BF is more than three times higher in adolescents with low VO_2peak_ [51,52].

The research hypothesis, assuming that for children with high BMI, the higher the fat mass in the body, the lower the level of cardiopulmonary capacity, has been confirmed. The study methodology that has been used was based on a measurement of direct oxygen absorption (CPET) and an analysis of the body mass composition (BIA method), and allows us to observe that the most essential component of the body mass correlated with VO_2peak_ level in the high BMI children population is fat-free mass.

In conclusion, the results obtained in this study have shown that the cardiopulmonary capacity of high-BMI children, expressed as absolute aerobic capacity and relative aerobic capacity is limited. Our findings suggest that it is not BMI or fat tissue mass, given at different rates (FM or BF%), but the fat-free mass that is the most correlated component of the body mass with cardiopulmonary capacity. Our research proves that using BIA as a method of body composition assessment is non-invasive, easy, cheap, quick (immediate results access), and allows the assessment of fat-free mass and fat mass in the body composition. The present study has shown that children with a high BMI correlate with body composition’s cardiopulmonary capacity component, with a fat-free mass. Therefore, this rate may be the most valuable for health care members, doctors, nutritionists, and physiotherapists to help monitor the body mass reduction in correlation with appropriate diet and physical activity.

Our study has some limitations. First, even though we analyzed high BMI children’s body compositions, the results were not analyzed. It comes with the fact that there is no reference data presenting the difficulty in describing the research results. Another limitation is the large but heterogeneous test population with a high BMI (19.30–36.00). This is because there is no consensus in the literature regarding the cutoff point of fat mass percentage in the total body mass in the pediatric population, which would allow the classification of appropriate reference groups. Additionally, this study aimed to determine the association between the individual parameters of cardiopulmonary capacity and selected parameters of body mass composition in children with high BMI. It justifies some deficiencies and suggests the necessity of a careful interpretation of the results.

Moreover, there are not many scientific publications on evaluating capacity, direct CPET, and body mass analysis of overweight school-age children. The strength of our study is the use of the examination method involving standardized exertion protocol to evaluate aerobic capacity and understandable criteria for reaching VO_2peak_/VO_2max_. The highest standards of comfort and safety were provided by the two sizes of the cycle ergometer and TANITA MS-780 MC scales.

## 5. Conclusions

The great attention of many medical specialists to our subject encourages the recognition of other relationships between body composition and cardiopulmonary capacity level, including age, sex, and a larger group of children with different BMIs. Despite the preceding limitations, based on the performed tests, the following conclusions are drawn:

1. In the case of children and youth with higher BMI, there is a strong correlation between the amount of fat-free mass in total body mass and cardiopulmonary capacity expressed as the peak amount of oxygen uptake (VO_2peak_) and the peak amount of oxygen uptake per 1 kg of body mass (VO_2peak/kg_).
2. The cross-sectional nature of the present study does not allow causal inferences to be made about the relationship between the measured variables. More longitudinal data and randomized controlled studies are urgently needed to determine the causality between cardiopulmonary capacity and body mass composition in children and adolescents with high body weight.
3. Body mass reduction among children with high BMI with the use of appropriate, correlated diet and physical activity needs to be monitored not only by BMI but also by fat and fat-free mass in total body mass, as well as by monitoring cardiopulmonary capacity level, especially during fast growth and maturation of a young individual.

## Figures and Tables

**Table 1:** Demographic and anthropometric parameters in the study group.

Parameters	Group (*n* = 100)		*p*-Value
Age (y); M ± SD, Min–Max	11.75 ± 1.93	7.17–15.91	0.50 *
Height (cm); M ± SD, Min–Max	154.00 ± 12.44	122.00–195.00	0.14 *
Weight (kg); Me, Q1–Q3	58.05	48.60–67.95	0.00
BMI percentile; Me, Q1–Q3	95.00	91.95–99.60	0.00
Z-score BMI; Me, Q1–Q3	1.74	1.40–2.62	0.00
BMI; Me, Q1–Q3	23.55	21.80–26.85	0.00

Indications: M, mean; SD, standard deviation; Me, median; Min, minimum value; Max, maximum value; Q1, lower quartile; Q3, upper quartile; Shapiro-Wilk test *p*-value. * *p* > 0.05 indicates the normal distribution of the data.

**Table 2:** Body mass composition parameters (Tanita test).

Body Mass Composition Parameters	Abbreviation	Definition	Unit
Body Mass Index	BMI	Quetelet II Rate; is a value derived from the mass (weight) and height;	kg/m^2^
Basal Metabolic Rate	BMR	it is the minimum amount of kJ needed to rest the body for 24 h to support basic life functions	kJ
Body Fat Percentage	BF%	Percentage of fat tissue in the body mass	%
Fat Mass	FM	Actual fat tissue mass	Kg
Fat-Free Mass	FFM	Fat-Free Mass	Kg
Total Body Water	TBW	Total amount of water in the body	%
Predicted Muscle Mass	MM	Predicted muscle mass includes skeletal muscle mass, smooth muscle mass, and water	Kg
Impedance	IMP	Body tissues’ electrical resistance	ohm
Bone Mass	BM	Predicted bone minerals mass	Kg
Phase angle	PA	Rate that determines health and cell integrity, is used to predict the nutritional value.	kHz

**Table 3:** Summary of data of body mass composition (Tanita test).

Parameters	Group (*n* = 100)		
	M ± SD	Min-Max	*p*-Value
BMR	6388.63 ± 1260.00	4551.00–9977.20	0.91 *
BF%	31.89 ± 5.65	13.10–48.70	0.97 *
FM	19.72 ± 7.63	7.60–43.00	0.88 *
FFM	39.89 ± 10.74	22.40–70.80	0.95 *
TBW	30.21 ± 8.56	16.90–51.80	0.94 *
PMM	38.44 ± 10.44	21.20–67.30	0.95 *
IMP	687.77 ± 105.06	451.99–956.77	0.97 *
BM	2.1 ± 0.54	1.20–3.50	0.94 *
PhA	5.46 ± 0.96	1.70–10.56	0.69 *

Indications: M, mean; SD, standard deviation; Min, minimum value; Max, maximum value; BMR, basal metabolic rate; BF%, body fat percentage; FM, fat mass; FFM, fat-free mass; TBW, total body water; PMM, predicted muscle mass; IMP, impedance; BM, bone mass; PhA, phase angle; Shapiro-Wilk test *p*-value. * *p* > 0.05 indicates the normal distribution of the data.

**Table 4:** Summary of data of cardiopulmonary capacity (CPET).

Parameters	Group (*n* = 100)		*p*-Value
HR; Me, Q1–Q3	184.00	170.00–192.00	0.00
BF; Me, Q1–Q3	41.00	36.00–51.00	0.00
VO_2peak_; Me, Q1–Q3	1.49	1.22–1.79	0.00
VO_2peak/kg_; Me, Q1–Q3	28.00	21.00–32.00	0.04
W; Me, Q1–Q3	99.00	72.50–130.00	0.00
VE; Me, Q1–Q3	49.30	41.05–60.05	0.00
VE/VO_2_; M ± SD, Min–Max	30.98 ± 4.56	21.90–42.30	0.11 *
VE/VCO_2_; Me, Q1–Q3	30.55	28.65–32.25	0.00
RER; M ± SD, Min–Max	1.00 ± 0.07	0.81–1.80	0.64 *

Indications: M, mean; SD, standard deviation; Me, median; Min, minimum value; Max, maximum value; Q1, lower quartile; Q3, upper quartile; HR, Heart Rate, BF, Breathing Frequency, VO_2peak_, peak oxygen consumption/maximal oxygen uptake; VO_2peak/kg_, VO_2peak_ per body mass; W, peak power output; VE, minute ventilation volume; VE/VO_2_, ventilation equivalent for oxygen; VE/VCO_2_, Ventilation equivalent for carbon dioxide; RER, Respiratory Exchange Ratio; Shapiro-Wilk test *p*-value. * *p* > 0.05 indicates the normal distribution of the data.

**Table 5:** Spearman correlation between BMI and particular parameters assessing body mass composition.

BMI	rho	*p*-Value
BF%	0.48	0.00 *
FM	0.83	0.00 *
FFM	0.74	0.00 *
PhA	0.37	0.06

Indications: * *p* < 0.05; rho, Spearman correlation; *p* value, statistical significance; BMI, body mass index; BF%, body fat percentage; FM, fat mass; FFM, fat-free mass; PhA, phase angle.

**Table 6:** Linear regression.

BMI as a Predictor of:	R^2^	F	*p*	Model Equation	R^2^
HR	0.0253	F (1,98) = 2.55	0.1136	HR = 196.65 − 0.69 × BMI	0.0253
BF	0.1089	F (1,98) = 11.98	0.0008 *	BF = 65.98 − 0.9055 × BMI	0.1089
VO_2peak_	0.1279	F (1,98) = 14.38	0.0003 *	VO_2peak_ = 0.34 + 0.0495 × BMI	0.1279
VO_2peak/kg_	0.0629	F (1,98) = 6.58	0.0118 *	VO_2peak/kg_ = 39.83 − 0.50 × BMI	0.0629
W	0.0001	F (1,98) = 0.01	0.9170	W = 110.50 − 0.13 × BMI	0.0001
VE	0.0181	F (1,98) = 1.80	0.1823	VE = 34.00 + 0.69 × BMI	0.0181
VE/VEO_2_	0.0047	F (1,98) = 0.46	0.4998	VE/VEO_2_ = 29.01 + 0.08 × BMI	0.0047
VE/VCO_2_	0.0126	F (1,98) = 1.25	0.2656	VE/VCO_2_ = 27.60 + 0.10 × BMI	0.0126
RER	0.0676	F (1,98) = 7.10	0.0090 *	RER = 1.116 − 0.00 × BMI	0.0676

Indications: * *p* < 0.05, HR, Heart Rate, BF, Breathing Frequency, VO_2peak_, peak oxygen consumption/maximal oxygen uptake; VO_2peak/kg,_ VO_2peak_ per body mass; W, peak power output; VE, minute ventilation volume; VE/VO_2_, ventilation equivalent for oxygen; VE/VCO_2_, Ventilation equivalent for carbon dioxide; RER, Respiratory Exchange Ratio.

**Table 7:** Spearman correlation between various parameters of body mass composition and capacity parameters.

CPET	BMI		BF%		FM		FFM		PhA	
	rho	*p*-Value	rho	*p*-Value	rho	*p*-Value	rho	*p*-Value	rho	*p*-Value
HR	−0.19	0.06	−0.08	0.42	−0.08	0.43	−0.01	0.95	−0.15	0.13
BF	−0.36	0.00 *	−0.10	0.30	−0.35	0.00 *	−0.33	0.00 *	−0.01	0.90
VO_2peak_	0.35	0.00 *	0.06	0.52	0.36	0.00 *	0.55	0.00 *	0.19	0.06
VO_2peak/kg_	−0.27	0.00 *	−0.22	0.03 *	−0.29	0.00 *	−0.13	0.19	0.06	0.57
W	0.04	0.67	−0.04	0.67	0.11	0.27	0.29	0.00 *	0.19	0.05
VE	0.14	0.15	0.03	0.76	0.20	0.04 *	0.38	0.00 *	0.15	0.15
VE/VEO_2_	0.02	0.87	0.12	0.25	0.06	0.53	0.04	0.68	−0.04	0.70
VE/VCO_2_	0.06	0.57	0.14	0.15	0.09	0.37	0.04	0.66	0.08	0.42
RER	−0.31	0.00 *	−0.15	0.13	−0.28	0.00 *	−0.18	0.08	−0.10	0.30

Indications: rho-Spearman correlation; * *p* < 0.05; BMI, Body Mass Index, BF%, Body Fat Percentage; FM, Fat Mass; FFM, Fat-Free Mass; PhA, Phase Angle; HR, Heart Rate, BF, Breathing Frequency, VO_2peak_, peak oxygen consumption/maximal oxygen uptake; VO_2peak/kg_, VO_2peak_ per body mass; W, peak power output; VE, minute ventilation volume; VE/VO_2_, ventilation equivalent for oxygen; VE/VCO_2_, Ventilation equivalent for carbon dioxide; RER, Respiratory Exchange Ratio.

## Data Availability

The data supporting the results of this study are available from the corresponding author upon reasonable request from any qualified investigator.
